# Supplementary material for: CRISPR/Cas9 Mediated Knockout of Cyclooxygenase-2 Gene Inhibits Invasiveness in A2058 Melanoma Cells
Source: Cells. 2022 Feb 21;11(4):749. doi: 10.3390/cells11040749 (PMC8870212; doi:10.3390/cells11040749)

cells

MDPI

Article

CRISPR/Cas9 mediated knockout of cyclooxygenase-2 gene inhibits invasiveness in A2058 melanoma cells

Cathleen Haase-Kohn [1*], Markus Laube [1], Cornelius K. Donat[2], Birgit Belter[1] and Jens Pietzsch [1,3]

Supporting Information

Content:

- S1: Original Western Blots of COX-2 protein content
- S2: Prostaglandin-E2 ELISA of A2058 and three clones of A2058-COX-2KO cells
- S3: In vitro cell growth of A2058 and three clones of A2058-COX-2KO cells
- S4: Subcutaneous tumor growth of two additionally A2058-COX-2KO cell lines
- S5: Representative images of the scratch assay in 10% FCS
- S6: Representative images of the scratch assay in 0% FCS

Figure S1: Protein content of COX-2 in A2058 and A2058-COX-2KO melanoma cell lines

TOP: β-actin
Cell lysates,
substrate Pico, exposure 30 s

Bottom: COX-2
Cell lysates,
substrate Pico:Femto 4:1,
exposure 300 s

ß-actin

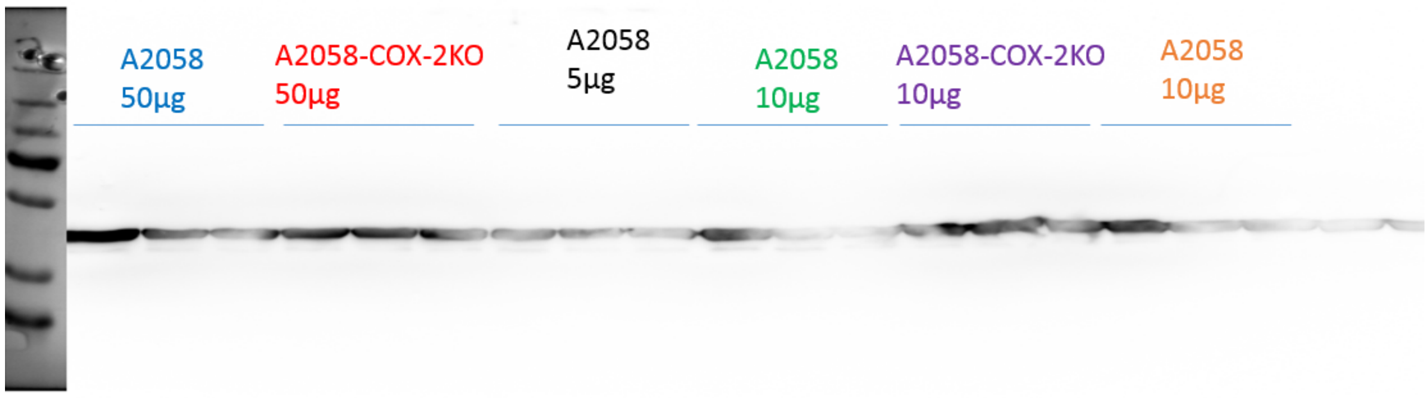

COX-2

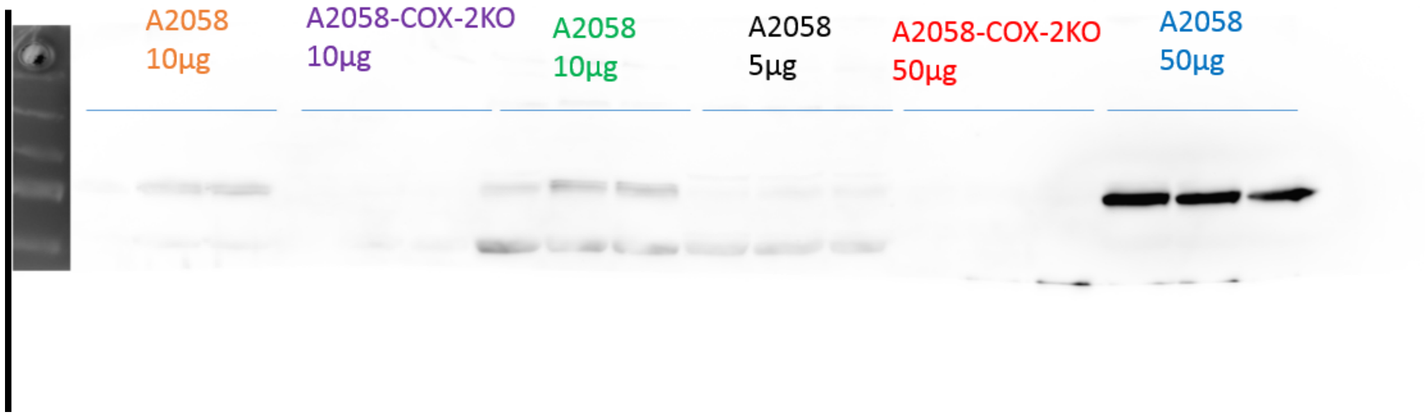

Figure S2: Prostaglandin E2 ELISA in cultured supernatants from A2058 and three different clones of A2058-COX-2KO cell lines in melanoma

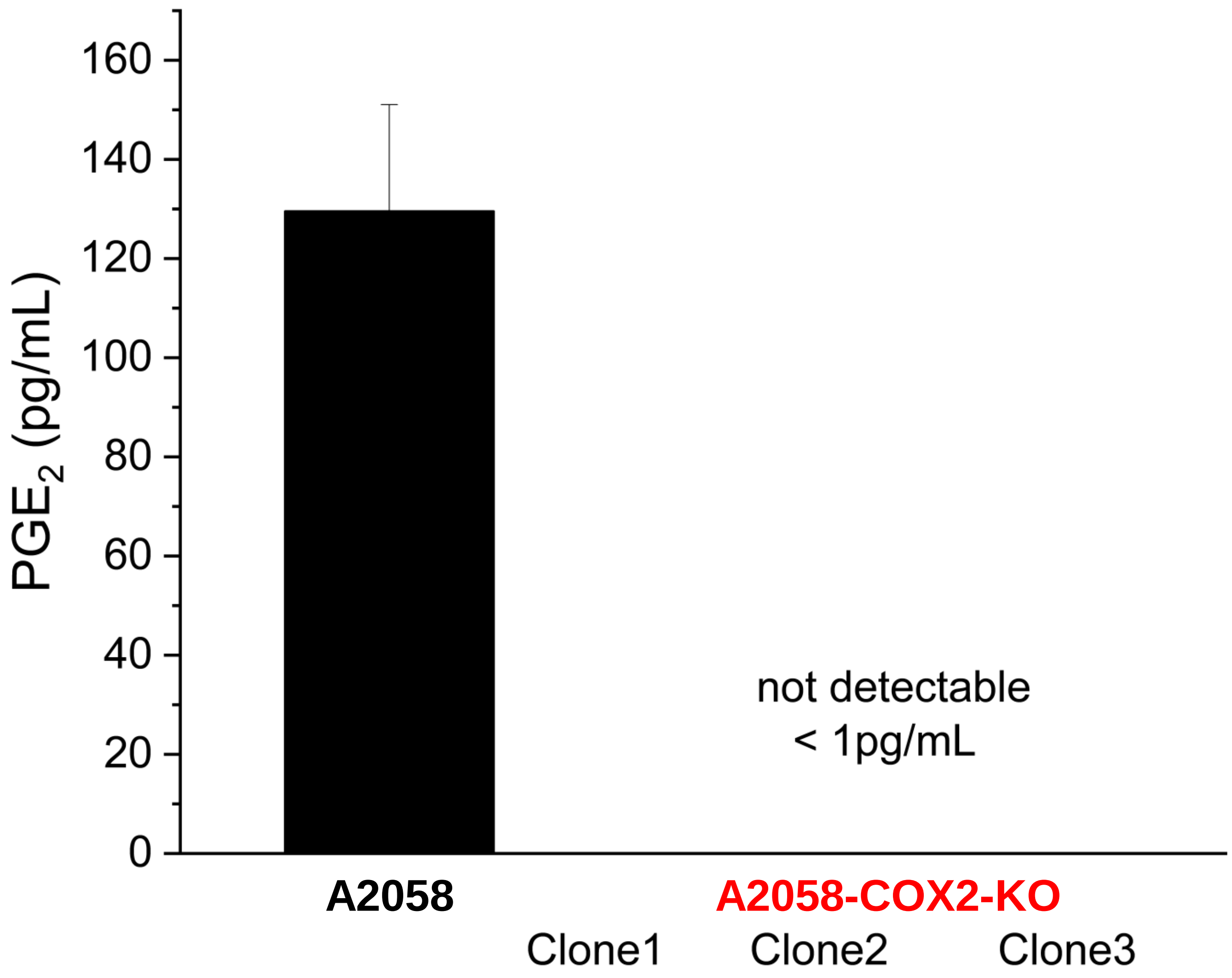

Figure S3: Analysis of cellular growth of A2058 and three different clones of A2058-COX-2KO (ACKO) cells using a CASY1 cell counter.

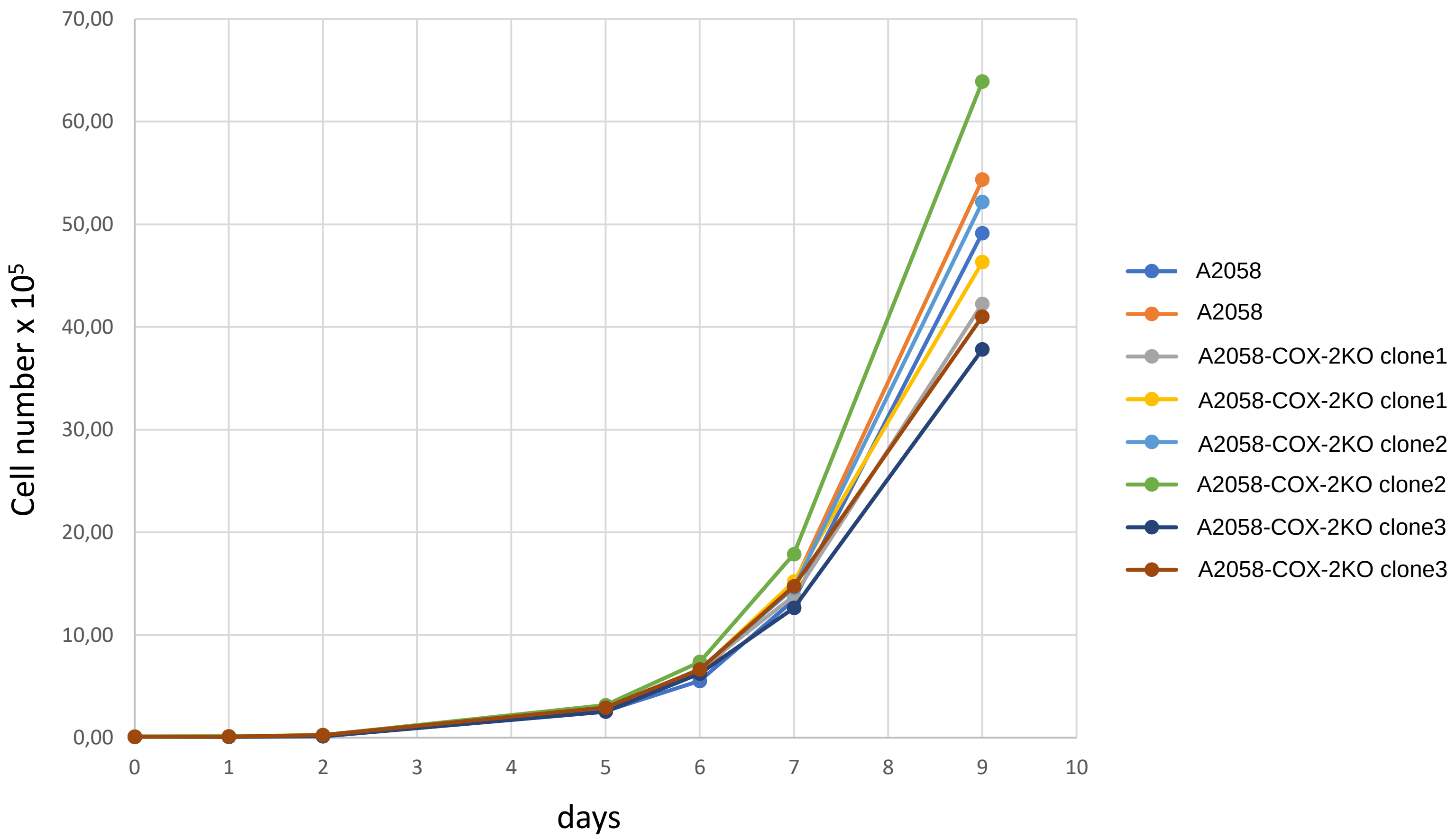

Figure S4: Tumor growth rate and progression analysis after the subcutaneous injection of two additional clones of A2058-COX-2KO cells in NMRI-nude mice.

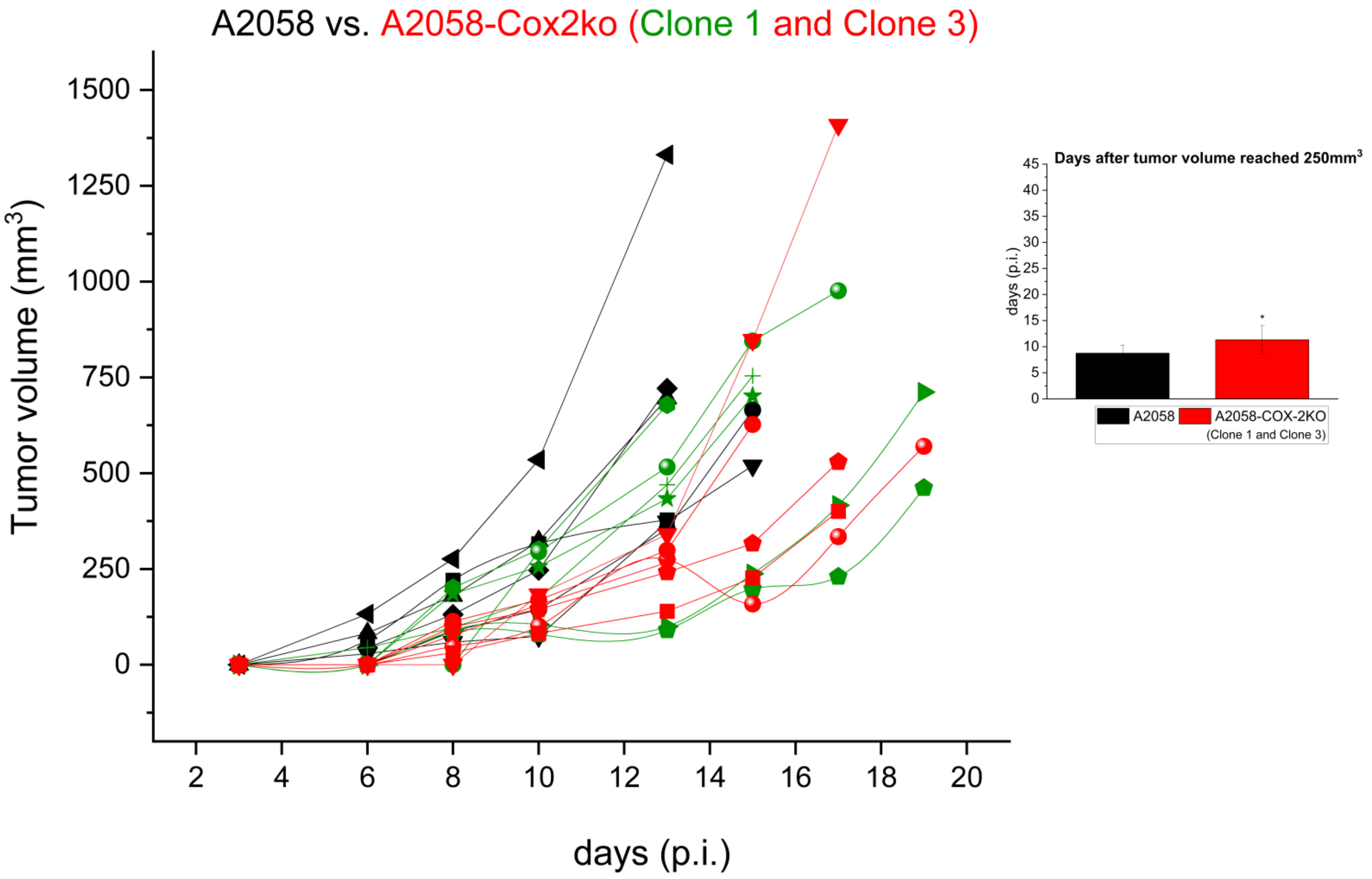

Figure S5: Representative images of scratch migration assay of A2058 and A2058-COX-2KO with 10% FCS.

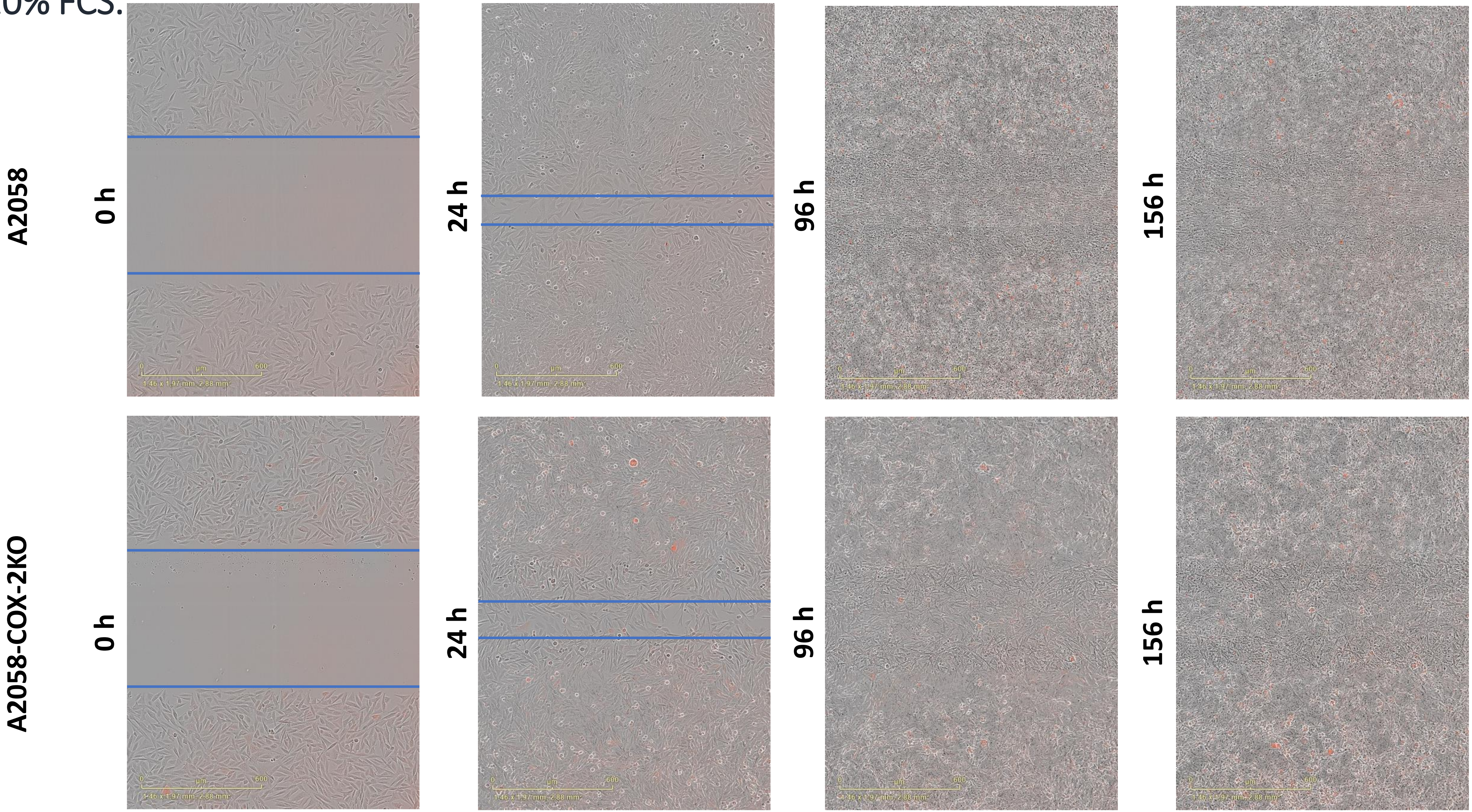

Figure S6: Representative images of scratch migration assay of A2058 and A2058-COX-2KO without FCS.

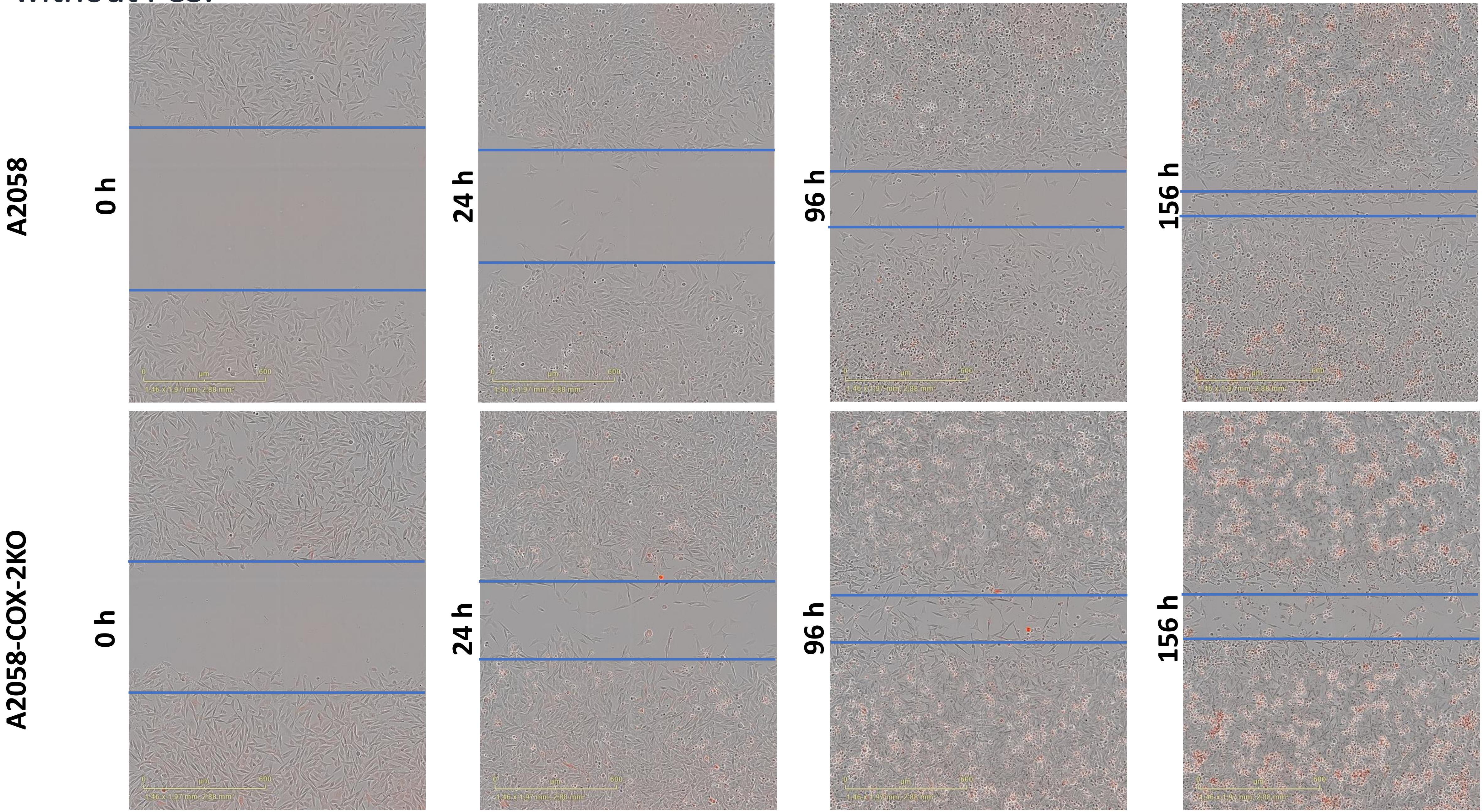

Supplement: Supplementary file 1 [file cells-11-00749-s001.zip › cells-1540577-supplementary..pdf]
